# BMP-2 gene delivery in cell-loaded and cell-free constructs for bone regeneration

**DOI:** 10.1371/journal.pone.0220028

**Published:** 2019-07-31

**Authors:** Loek D. Loozen, Moyo C. Kruyt, Angela H. M. Kragten, Ted Schoenfeldt, Michiel Croes, Cumhur F. Oner, Wouter J. A. Dhert, Jacqueline Alblas

**Affiliations:** 1 Dept. Orthopaedics, University Medical Center Utrecht, Utrecht, The Netherlands; 2 Faculty of Veterinary Medicine, Utrecht University, Utrecht, The Netherlands; Università degli Studi della Campania, ITALY

## Abstract

To induce osteogenicity in bone graft substitutes, plasmid-based expression of BMP-2 (pBMP-2) has been successfully applied in gene activated matrices based on alginate polymer constructs. Here, we investigated whether cell seeding is necessary for non-viral BMP-2 gene expression *in vivo*. Furthermore, to gain insight in the role of BMP-producing cells, we compared inclusion of bone progenitor cells with non-osteogenic target cells in gene delivery constructs. Plasmid DNA encoding GFP (pGFP) was used to trace transfection of host tissue cells and seeded cells in a rat model. Transgene expression was followed in both cell-free alginate-ceramic constructs as well as constructs seeded with syngeneic fibroblasts or multipotent mesenchymal stromal cells (MSCs). Titration of pGFP revealed that the highest pGFP dose resulted in frequent presence of positive host cells in the constructs. Both cell-loaded groups were associated with transgene expression, most effectively in the MSC-loaded constructs. Subsequently, we investigated effectiveness of cell-free and cell-loaded alginate-ceramic constructs with pBMP-2 to induce bone formation. Local BMP-2 production was found in all groups containing BMP-2 plasmid DNA, and was most pronounced in the groups with MSCs transfected with high concentration pBMP-2. Bone formation was only apparent in the recombinant protein BMP-2 group. In conclusion, we show that non-viral gene delivery of BMP-2 is a potentially effective way to induce transgene expression *in vivo*, both in cell-seeded as well as cell-free conditions. However, alginate-based gene delivery of BMP-2 to host cells or seeded cells did not result in protein levels adequate for bone formation in this setting, calling for more reliable scaffold compatible transfection methods.

## Introduction

Cell-based strategies for bone regeneration have been extensively investigated [1, 2] and the applicability of multipotent mesenchymal stromal cells (MSCs) has been demonstrated in several preclinical models [3]. Despite this achievement, translation to clinical practice appears to be difficult and only few clinical studies have been reported, which consisted mainly of case series. Main limiting factors when upscaling the technique include metabolic stress due to hypoxia and lack of early vascularization and activation of the host immune system [4, 5]. Until now the precise fate of implanted cells is not clear. Tracing studies have shown contradictory results for long-term engraftment of MSCs [6]. The use of MSCs for bone regeneration has not been adopted in clinical practice but is regarded as a feasible option with several clinical trials ongoing [7, 8].

To induce bone in clinical situations, growth factor-based strategies have been more frequently applied than cell-based applications. Members of the TGF-β superfamily of growth factors, such as bone morphogenetic proteins (BMPs) have been successfully used in spinal fusion surgery and tibial fracture healing [9–11]. In order to achieve clinically meaningful effects, BMP-2 is applied on a collagen sponge at a dosage that is much higher than the minimum effective dose to compensate for wash-out and rapid degradation by proteinases [12]. Despite this supraphysiological dose, BMP-2 remains present for only a few days [13]. BMP-2 use is associated with serious complications probably due to these supraphysiological doses [11, 14]. In natural circumstances, during uncomplicated fracture healing, the BMP-2 levels are elevated for up to three to four weeks [15, 16]. This supports the strive for therapies associated with a sustained and more physiological BMP-2 release to reduce the risk of adverse effects [17, 18]. Gene delivery is one of the approaches that enables BMP-2 production over a period of several weeks [19].

Gene delivery strategies consist of viral and non-viral methods. For bone regeneration non-viral transfection strategies are preferred over virus-based methods as they are considered to be safer. Immune responses or complications as a result of mutagenesis have not been described in clinical trials, even at high plasmid DNA dosages [20]. Furthermore, the transient nature of plasmid-based transgene expression is desirable in bone regeneration, as only a temporary BMP production is required. The main limitation of non-viral strategies is the transfection efficiency seen for most methods. The resulting low amounts of transgene expression in the target area have hampered clinical translation.

The combination of local gene delivery with the implantation of cells, as a target for the gene delivery, has been proven successful in preclinical models [21]. For this purpose, MSCs have been used most frequently as they are efficiently expanded, transfected and implanted [19]. Other attractive cell sources that can be harvested and re-implanted during surgery are bone marrow mononuclear cells or fat tissue-derived cells [22, 23]. The fate of these implanted transfected cells is however still unclear. Only a few studies showed long-term engraftment of donor cells via genetic tracing [24, 25]. In general, donor cells are rarely found in the regenerated tissue [26]. It remains unclear whether the levels and duration of BMP-2 protein production, which are reached by non-viral gene therapy, are sufficient for osteogenesis. Cell-free plasmid-based gene delivery, which omits the need for the steps of cell isolation, transfection and implantation targets the host cells for transgene expression and might be an elegant alternative [27].

In previous work, we developed a non-viral gene therapy technique using alginate hydrogel loaded with plasmid DNA containing the human BMP-2 coding region (pBMP-2) to transfect MSCs. The alginate hydrogel functions both as a delivery vehicle for plasmid DNA to cells [28, 29], and as temporary matrix for seeded cells [30, 31], making alginate a promising gene activated matrix (GAM) [29, 32]. Using this gene delivery approach, MSC-seeded constructs have resulted in detectable protein levels *in vitro* and abundant bone formation in mice [21]. To date, the role of transfected MSCs in relation to bone formation remains unclear. Other cell types have not been investigated to date and a single cell-free approach failed to show an increased bone formation [33]. This could be due to suboptimal dosage of pDNA, which has shown to be of great importance to transfection efficiency in other work [32].

In this study, we investigated the conditions for optimal transgene expression and secondly addressed whether cell seeding is indispensable for non-viral BMP-2 gene expression *in vivo*. Furthermore, because MSCs are BMP-2 responsive and will also produce BMP-2 secreted by neighboring cells, we compared bone progenitor cells with non-osteogenic producer cells (i.e. fibroblasts) in plasmid-based gene delivery constructs.

## Materials and methods

### Scaffold components

The ceramic scaffold was composed of porous biphasic calcium phosphate (BCP, Xpand biotechnology, Bilthoven the Netherlands) that was used in previous gene delivery studies [32, 33]. The 3x3x6 mm scaffolds consisted of 80±5% (w/v) hydroxyapatite and 20±5% (w/v) β-tricalcium phosphate which were 70±5% porous. The pore size was 200–800 μm. BCP scaffolds were cleaned in ultrasonic baths and autoclaved and subsequently used without further treatment.

Autoclaved high-viscosity alginate powder (International Specialty Products, ISP, Memmingen, Germany) was dissolved in alpha minimum essential medium (α-MEM, Gibco, Breda, The Netherlands) and used at a final concentration of 10 mg/ml, as previously described [32]. Autoclaving of alginate results in poorer gelation capacities, and may arguably decrease the molecular weight. Comparing different sterilization methods, including UV light, we found that autoclaving keeps the transfection efficiency unharmed, while resulting in full sterility.

### Plasmid DNA containing the GFP and BMP-2 genes

**Fig 1:**
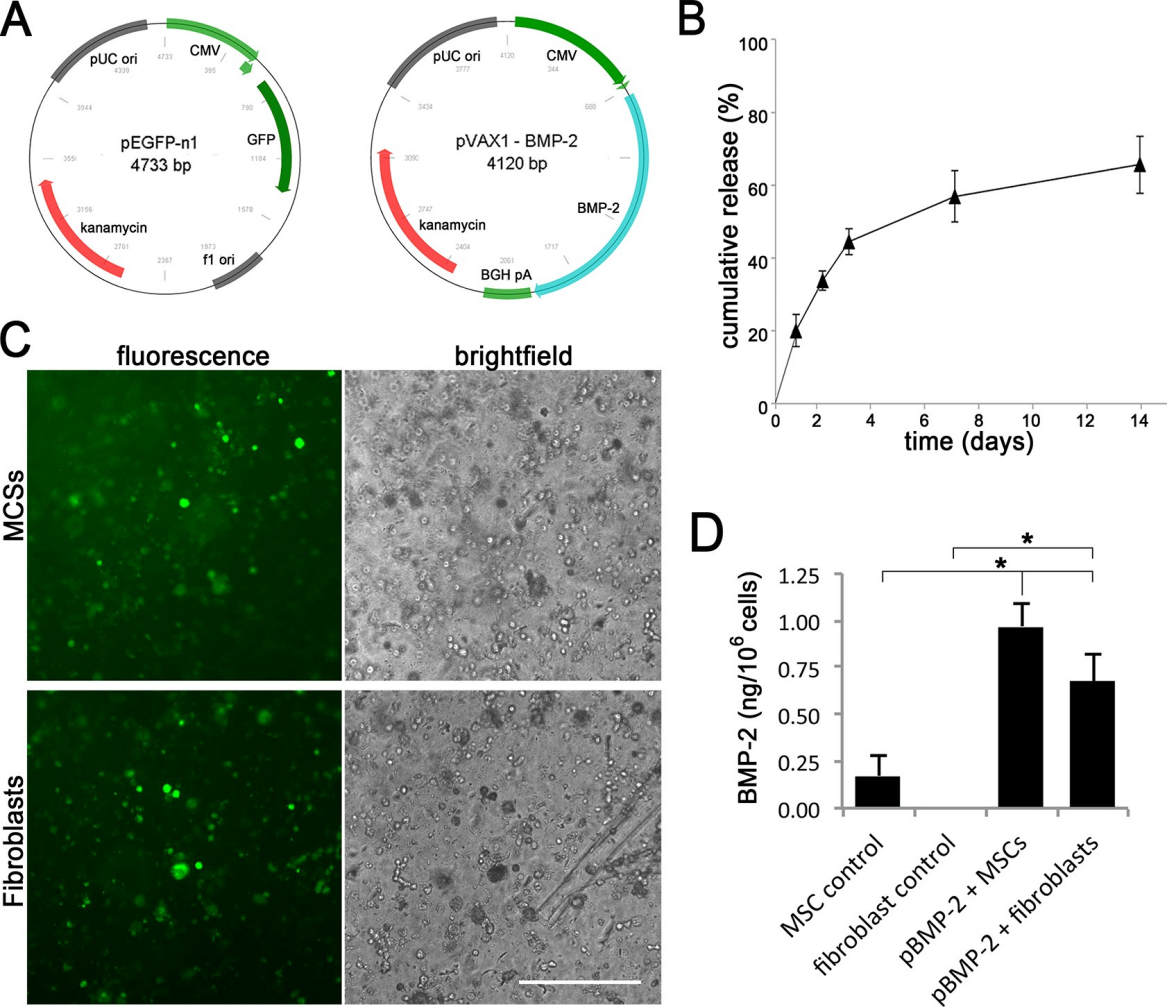
Plasmid retention and transgene expression in vitro. A) Vector maps of the pGFP and pBMP-2. B) In vitro plasmid DNA release over 14 days from constructs consisting of BCP scaffold, alginate hydrogel and pBMP-2. Initial loading concentration was 100 μg/ml. Data are shown from a single experiment (technical triplicate), which was repeated with similar results. C) Microscopic images of pGFP transfected MSCs as well as fibroblasts after 7 days incubation in alginate. Transfection efficiency was 21 ± 4% for the MSC group and 16 ± 3% fibroblasts group (controls not shown). Representative images are shown for two individual experiments. Scale bar = 200 μm. D) BMP-2 protein release from constructs containing MSCs and fibroblasts transfected with pBMP-2 (100 μg/ml) and empty control DNA at day 7. Results represent mean ± SD of technical triplicates, the experiment was repeated with similar results. * = p < 0. 05. Original data of graph B and D can be found in Supporting information S1 and S2 Data.

Vectors consisted of pEGFP-N1 (BD Biosciences, Franklin Lakes, NJ, USA), pVAX1/rhBMP-2 (Fig 1A) and control vector was pVAX1 (Invitrogen) without insert. The pBMP-2 construct contained the full-length human recombinant BMP-2 cDNA. Plasmid DNA was isolated, purified and cleared from endotoxins (EndoFree Plasmid Maxi kit, Qiagen K.K., Tokyo, Japan).

### Isolation of fibroblasts and MSCs

Fibroblasts were isolated from skin samples, collected and pooled from male Fischer 344 inbred rats (Charles River Laboratories, France). Fur was shaved and skin was disinfected with 70% ethanol. A 1 cm^2^ skin sample was obtained aseptically and cut in smaller fragments of approximately 1 mm^2^. These fragments were then digested by treatment with a collagenase solution over 30 minutes under constant stirring. The solution consisted of 0.14 Wunsch units/mL Liberase Blendzyme 3 (Sigma) in culture medium (DMEM/F12 (Gibco), 100 U/ml penicillin, 100 μg/ml streptomycin, 50 mg/mL gentamicin and 0.5 mg/mL fungizone (Sigma)). Subsequently cells were pelleted and washed in DMEM/F12 with 10% (v/v) fetal calf serum (Cambrex, Charles City, IA, USA), antibiotics and fungizone as described above. Thereafter cells were cultured in fibroblast expansion medium (DMEM/F12 with 10% (v/v) fetal calf serum, 100 U/ml Penicillin and 100 μg/ml Streptomycin) at 37°C and 5% CO_2_ in a humidified incubator.

MSCs were harvested and pooled from the marrow of tibias and femurs of the same animals. The bones were aseptically removed and placed in PBS. The epiphyses were cut, while the diaphyses were pierced with a sterile 16-gauge needle. Subsequently the marrow was flushed out with 5 mL PBS. The collected marrow was filtered, pelleted and resuspended in MSC expansion medium (DMEM, 10% (v/v) fetal calf serum, 100 U/ml Penicillin and 100 μg/ml Streptomycin) and cultured at 37°C and 5% CO_2_ in a humidified incubator.

### Construct composition

A gene tracing study lasting two weeks was performed in rats using pGFP at different concentrations, with or without cells as detailed in Table 1. Osteogenicity of plasmid-expressed pBMP-2 was studied in experiment in rats lasting eight weeks as outlined in Table 2. An additional group was added with a 5-fold higher pDNA concentration compared to the gene tracing study because a plateau concentration was not reached yet and high dosages of pDNA were not disadvantageous for bone formation previously [34]. BMP-2 protein at a final concentration of 25 μg/ml was used as the positive control.

**Table 1:** Experimental groups for gene tracing study using pGFP (two weeks in rats).

Group	Ceramic	Alginate	Cells	pEGFP-n1	pVAX1	Location
pGFP	+	+	-	100 μg	-	i.m., s.c. and orthotopic
pGFP	+	+	-	10 μg	-	i.m. & s.c.
pGFP	+	+	-	1 μg	-	i.m. & s.c.
pGFP + fibroblasts	+	+	10^7^	100 μg	-	i.m. & s.c.
pGFP + MSCs	+	+	10^7^	100 μg	-	i.m. & s.c.
Control	+	+	-	-	100 μg	i.m. & s.c.

All component concentrations are per ml alginate; i.m.: intramuscular implant (n = 6); s.c.: subcutaneous implant (n = 6).

**Table 2:** Experimental groups used to assess osteogenicity of plasmid based expression of pBMP-2 (eight weeks in rats).

Group	Ceramic	Alginate	Cells	pVAX1-BMP-2	pVAX1	rhBMP-2	Location
pBMP-2	+	+	-	500 μg	-	-	i.m.
pBMP-2	+	+	-	100 μg	-	-	i.m.
pBMP-2 + fibroblasts	+	+	10^7^	100 μg	-	-	i.m.
pBMP-2 + MSCs	+	+	10^7^	100 μg	-	-	i.m.
rhBMP-2	+	+	-	-	-	25 μg	i.m.
Control	+	+	-	-	100 μg	-	i.m.
Fibroblast control	+	+	10^7^	-	100 μg	-	i.m.
MSC control	+	+	10^7^	-	100 μg	-	i.m.

All component concentrations are per ml alginate; i.m.: intramuscular implant (n = 14).

Cryopreserved fibroblasts and MSCs were thawed, washed and cultured for three days in expansion medium. Cells were detached using 2 mL of 0. 25% (v/v) trypsin solution (Sigma), washed and resuspended at 10^7^ cells/ml in alginate (10 mg/ml). Gene-activated matrices were created by adding pGFP or empty control plasmid to the alginate, with and without cells, according to the groups in Tables 1 and 2. The alginate mixtures were then carefully pipetted on the porous BCP scaffolds. Even distribution of cells and alginate throughout the constructs was ensured by visual inspection and microscopy prior to implantation. A total volume of 40 μl supplemented alginate was added to each BCP scaffold. To polymerize the alginate, the constructs were submerged in 100 mM aqueous CaCl_2_ supplemented with 10 mM of 4-(2-hydroxyethyl)-1-piperazineethanesulfonic acid (HEPES, pH 7.4). After 10 minutes the Ca-solution was replaced by MSC expansion medium.

### *In vitro* experiments

To assess the *in vitro* plasmid DNA release profiles, constructs were made consisting of BCP scaffold, alginate hydrogel and pBMP (100 μg/ml). These constructs were kept in PBS (500 μl per sample) at 37°C in a humidified incubator for 14 days. Samples were taken at day 1, 2, 3, 7 and 14. The pDNA was separated from other DNA fragments via agarose gel (1% w/v) electrophoresis containing 0.5 μg/ml EtBr (Sigma). EtBr intensity of the plasmid DNA bands was quantified via pixel comparison with a calibration curve made with known concentrations of the same pDNA.

To demonstrate alginate-mediated transfection, fibroblasts or MSCs (10^7^ cells/ml) and pGFP or empty control plasmid (100 μg/ml) were suspended in 100 μl alginate (10 mg/ml) plugs without BCP scaffold. Plugs were polymerized and kept at 37°C in a humidified incubator, and the medium was changed every three days. At day 7, GFP expression was assessed using an inverted fluorescence microscope (AMG EVOS, Fisher).

To quantify the efficiency of BMP-2 synthesis, fibroblasts or MSCs (10^7^ cells/ml) and pBMP-2 or empty control plasmid (100 μg/ml) were suspended in 100 μl alginate (10 mg/ml) plugs without BCP scaffold. Constructs were kept at 37°C in 2 ml expansion medium in a humidified incubator. At day 7 the gel was depolymerized with citrate buffer (150 mM NaCl, 55 mM sodium citrate and 20 mM EDTA in H_2_O) for 15 minutes at room temperature. The BMP-2 concentration in the buffer and the medium was measured with ELISA (Quantikine #DBP200 from R&D Systems) following standard protocol.

### Animals and implantation

After approval of the local animal care committee, 14 male Fischer 344 inbred rats (126–150 g; Charles River Laboratories, L’Arbresle, France) were used. Food and water were given ad libitum. The laboratory animal welfare officer monitored the animals’ general health and care. All procedures were performed under general anesthesia by inhalation of isoflurane and the incisions were closed using a vicryl 5–0 suture. Pain relief was given by subcutaneous injections of buprenorphine (Temgesic; Schering-Plough, Utrecht, The Netherlands), 0.05 mg/kg body weight, every 8h until 48h postoperatively. After shaving and disinfecting the back of the rats, a posterior midline incision was made to expose the paraspinal muscles. For the gene tracing study (groups outlined in Table 1), six subcutaneous and six paraspinal intramuscular pockets were made. In addition, one orthotopic spinous process defect location was created at the lumbosacral junction. After two weeks the animals were euthanized with CO_2_ asphyxiation and constructs were retrieved and processed for assessment of gene expression. For the pBMP-2 expression study (groups in Table 2) ten paraspinal intramuscular pockets were made per animal, of which eight were used for this study. In both studies, all groups were implanted according to a randomized block design. After eight weeks the animals were euthanized and constructs were retrieved and processed for assessment of bone formation.

### Post-mortem sample acquisition and processing

After retrieval, samples were fixed in 4% (w/v) formalin and processed for 5 μm thick decalcified paraffin sections through 0.5 M EDTA and alcohol dehydration series for GFP immunohistochemistry and hematoxylin and eosin (HE) staining. For the pBMP-2 expression study: after retrieval, samples were fixed and cut in half. One half was processed for paraffin embedding as described above for BMP-2 immunohistochemistry, tartrate-resistant acid phosphatase staining and HE staining. The other half was embedded in poly-methyl-methacrylate (PMMA), for undecalcified histology. Sections were stained with methylene blue and basic fuchsin.

### GFP immunohistochemistry

Immunohistochemistry was used to demonstrate GFP presence, as its intrinsic fluorescence was lost during the tissue processing. Paraffin sections were rehydrated, permeabilized with 0.1% Triton X-100 in PBS and incubated in 0.3% (v/v) H_2_O_2_ for 10 min and in 5% (w/v) bovine serum albumin (BSA) in PBS for 30 min. Sections were incubated for two hours at room temperature with chicken anti-GFP (ab13970, Abcam) at 20 μg/ml in PBS/5% BSA and subsequently washed and incubated with goat anti-chicken Alexa Fluor 594 (ab150176, Abcam) at 8 μg/ml in PBS/5% BSA for 30 min at room temperature. Nuclei were stained with DAPI and sections were mounted in aquamount. GFP presence was assessed with a fluorescence microscope (E600, Nikon). Per sample a midsection was stained and three pre-designated fields of view (ROI) were photographed. Two observers scored the blinded specimens on a scale from 0–3 corresponding to the number of positive cells per ROI (0 = negative; 1 = 1–5 GFP positive cells; 2 = 6–20 GFP positive cells; 3 = >20 GFP positive cells). Mean scores of both observers were compared. When the scores differed, agreement was reached after discussion.

### BMP-2 immunohistochemistry

BMP-2 protein detection was performed as described before [33]. Per sample, a midsection was stained and three pre-designated ROIs were photographed, a representative image was chosen. In short, paraffin sections were rehydrated and subsequently permeabilized with 0.1% Triton X-100 in PBS and then incubated in 0.3% (v/v) H_2_O_2_ for 10 min. Antigen retrieval steps were performed in 10 mM sodium citrate buffer (pH 6.0) for 30 min at 95°C. The sections were then incubated in 5% (w/v) bovine serum albumin (BSA) in PBS for 30 min and subsequently for two hours at room temperature with rabbit-anti-BMP-2 (C43125, LifeSpan BioSciences, Province, RI, USA) at 2.5 μg/ml in PBS/5% BSA. Control stainings were performed with non-immunized rabbit immunoglobulin (IgG) 2.5 μg/ml in PBS (Dako, X0903, Glostrup, Denmark). Subsequently samples were incubated with goat-anti-rabbit horseradish peroxidase (Dako, P0448, Glostrup, Denmark) at 1 μg/ml in PBS/5% BSA for 60 min at room temperature. The staining was developed with 3,3’-diaminobenzidine. Counterstaining was performed with Mayer’s hematoxylin.

### Tartrate-resistant acid phosphatase

Osteoclasts, macrophages, and dendritic cells expressing tartrate-resistant acid phosphatase (TRAP) were visualized using the Kit 386A-1KT (Sigma–Aldrich) according to the manufacturer’s instructions on decalcified paraffin sections [35]. TRAP-positive cells appear in red.

### Statistics

The statistical significance of differences between experimental groups in Fig 1D was assessed using the two-factor ANOVA and Tukey post-hoc test. Data are represented as mean ± standard deviation. P values <0.05 are considered statistically significant.

## Results

### *In vitro* assessment of construct performance

The release of pDNA to the surrounding environment was assessed to estimate release kinetics of pDNA from the constructs, which would allow invading and surrounding cells to be transfected after the implantation phase. Up to 50% of the initially loaded plasmid DNA was released in the first three days, which increased to more than 60% after two weeks (Fig 1B). This implies a prolonged availability of plasmid DNA for seeded cells but also for tissue-resident cells.

To demonstrate transfection of cells by the alginate GAM, fibroblasts or MSCs were included in alginate constructs with pGFP and assessed for *in vitro* GFP expression. GFP-positive cells were observed in both the fibroblast and MSC containing groups (Fig 1C) but were absent from the control samples (containing empty plasmid, results not shown).

To quantify BMP-2 production as a result of gene transfer, both cell types were transfected with pBMP-2 and cumulative production was measured at day 7. Unpublished work has shown that at this time point gene expression is optimal. Transfection of MSCs resulted in higher BMP-2 production than the other conditions, although fibroblasts also produced a substantial amount of BMP-2. In the MSC control group (transfected with empty plasmid) low levels of endogenously produced BMP-2 were measured. Note that the ELISA detects human BMP-2 as well as endogenous rat BMP-2. In previous studies we have shown that the produced BMP-2 is biologically active [32].

### Histological and immunological aspects of the implants

**Fig 2:**
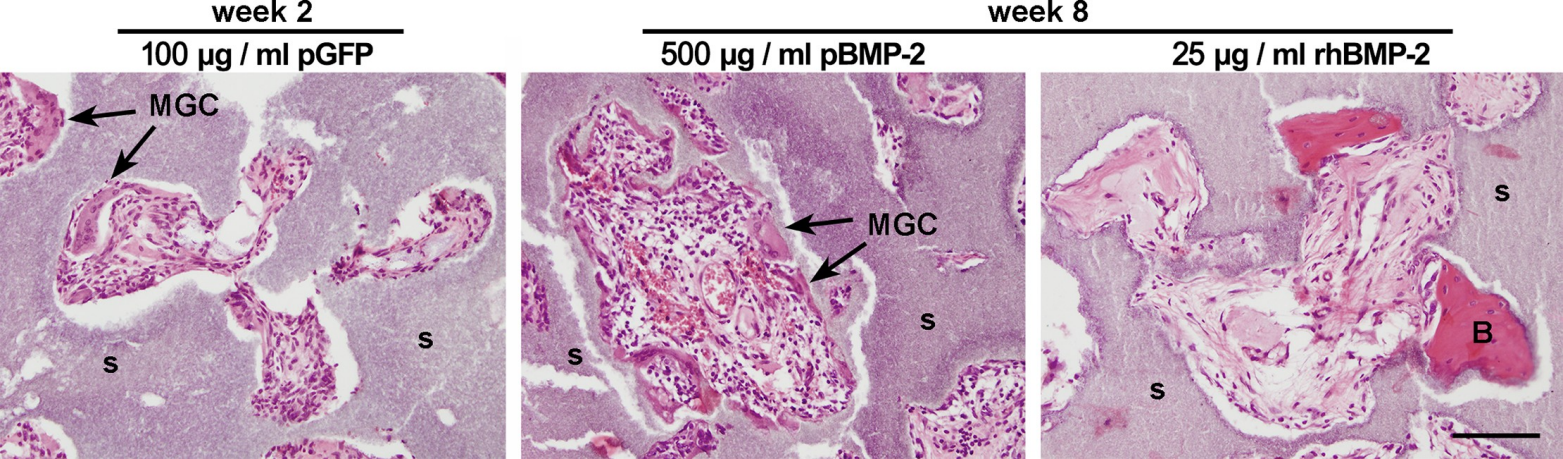
Tissue ingrowth and bone formation after two and eight weeks in vivo. The images show H&E-stained sections of i.m. samples, containing 100 μg/ml pGFP (week 2), 500 μg/ml pBMP-2 or 25 μg/ml rhBMP-2 (week 8) without seeded cells. A representative image of each group is shown. After two weeks, complete ingrowth of tissue could be observed as well as frequent presence of multinucleated giant cells (MGC). After eight weeks, alginate was no longer detectable in any of the constructs. In all groups containing pDNA a high cell density could be seen together with the frequent presence of MGCs. s = scaffold; B = bone; MGC = multinucleated giant cell. Scale bar = 100 μm.

No surgical complications occurred in the rats, and all samples were uneventfully retrieved. To evaluate the host response against the implants as well as infiltration of host cells, immunohistochemistry on decalcified tissue was done. Microscopic analysis of the pGFP tracing study after two weeks revealed tissue ingrowth with connective tissue throughout the entire scaffold, seen for every group and in all three implant locations. Distinct differences in cell morphology or cell density were not found. Multinucleated giant cells (MGCs) were frequently observed in all groups (Fig 2). Microscopic analysis after eight weeks of the pBMP-2 expression study showed extensive tissue ingrowth throughout the entire scaffold in all groups. Sporadically MGCs were present in the tissue, more evident in the groups with pDNA than the corresponding controls (Fig 2). Lymphoid clusters or fibrous capsules were not observed.

### Transgene expression in cell-free and cell-seeded implants

**Fig 3:**
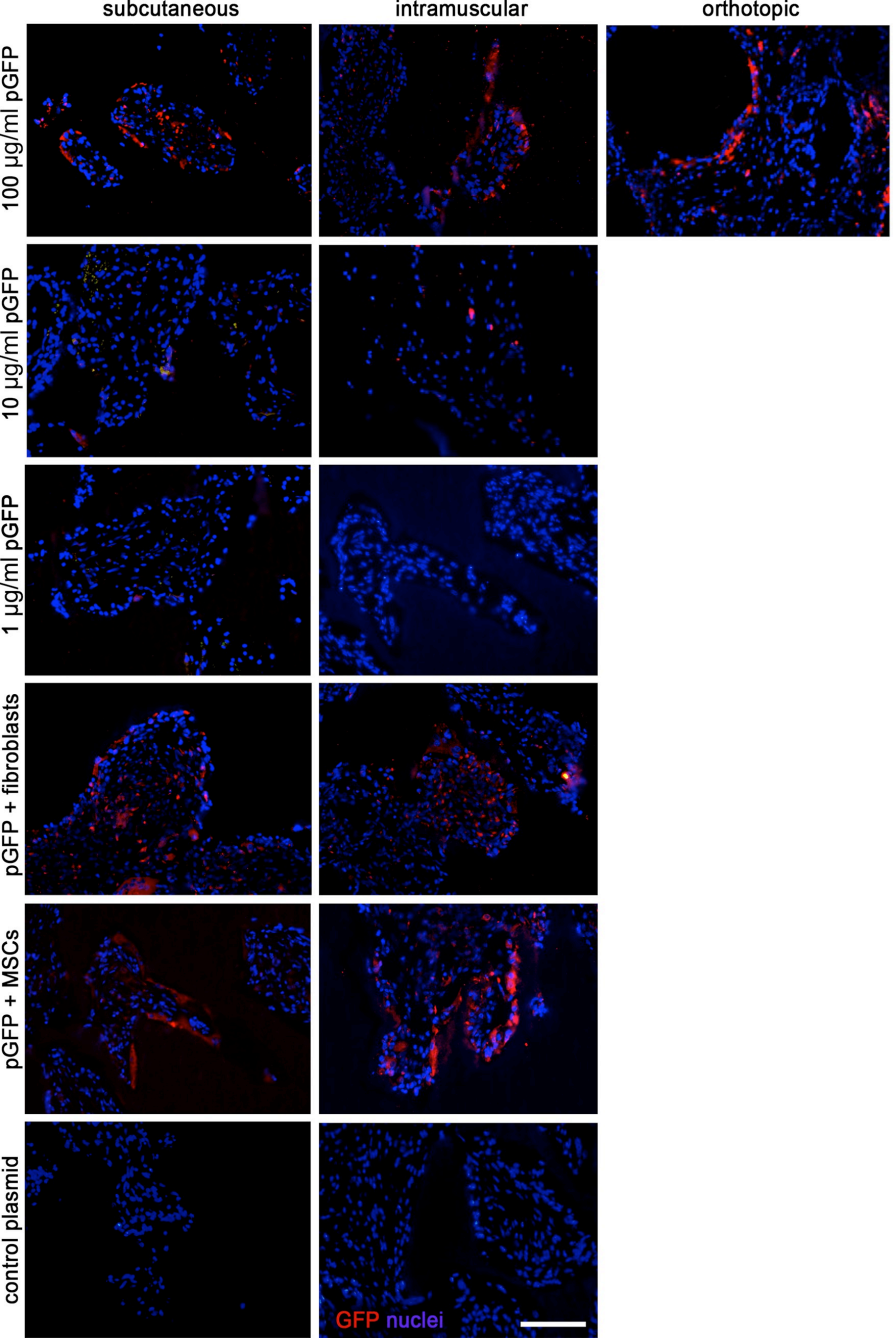
Qualitative imaging of in vivo gene expression. A representative image of the GFP immunohistochemistry (in red) for each group implanted in the subcutaneous (left column panels), intramuscular (middle panels) or orthotopic (right panel) locations. Each row represents an experimental group, as outlined in Table 2. Nuclei are DAPI stained (in blue). Scale bar = 100 μm for all panels.

Presence of GFP expression as a result of transfection was assessed with a semi-quantitative scoring on 4 animals. The results varied with respect to the type of cell being transfected, and was influenced by pDNA dosage and the presence of seeded cells. The two week implantation period was chosen based on in vitro work, and the notion that in vivo degradation and thereby plasmid release would be faster than in vitro. Fig 3 and Table 3 show representative images and quantified data respectively. It appears that for the cell-free groups the pGFP concentration of 100 μg/ml clearly resulted in abundant GFP transgene expression. The groups containing seeded cells, both fibroblasts and MSCs, also showed frequent expression of GFP. Overall observations of all samples indicated that GFP was mainly present within the construct, but in two intramuscular samples (100 μg/ml pGFP without seeded cells) tissue outside the construct boundaries stained positive as well. Intramuscular implants showed overall frequent transgene-positive cells. The orthotopic location was also associated with frequent GFP-positive cells. The control samples did not show any GFP, neither within the construct boundaries nor in the surrounding tissue.

**Table 3:** Scoring of transgene expression at the different implant locations.

	Rat 1			Rat 2			Rat 3			Rat 4			Sum of scores		
	i.m.	s.c.	ort.	i.m.	s.c.	ort.	i.m.	s.c.	ort.	i.m.	s.c.	ort.	i.m.	s.c.	ort.
pGFP 100 μg/ml	2	0	1	2	0	3	2	1	3	0	1	1	**6**	**2**	**8**
pGFP 10 μg/ml	0	0		1	0		0	0		0	0		**1**	**0**	
pGFP 1 μg/ml	0	0		0	0		0	0		0	0		**0**	**0**	
pGFP + fibroblasts	2	2		1	1		0	1		2	0		**5**	**4**	
pGFP + MSCs	1	1		3	0		3	1		2	3		**9**	**5**	
Control	0	0		0	0		0	0		0	0		**0**	**0**	

Each implant was given a score (0, 1, 2 or 3) for the amount of GFP expression (as detailed in the M&M section). The sum of the total scores per group is depicted. i.m.: intramuscular; s.c.: subcutaneous; ort.: orthotopic.

### Bone formation and assessment of BMP-2 presence

**Fig 4:**
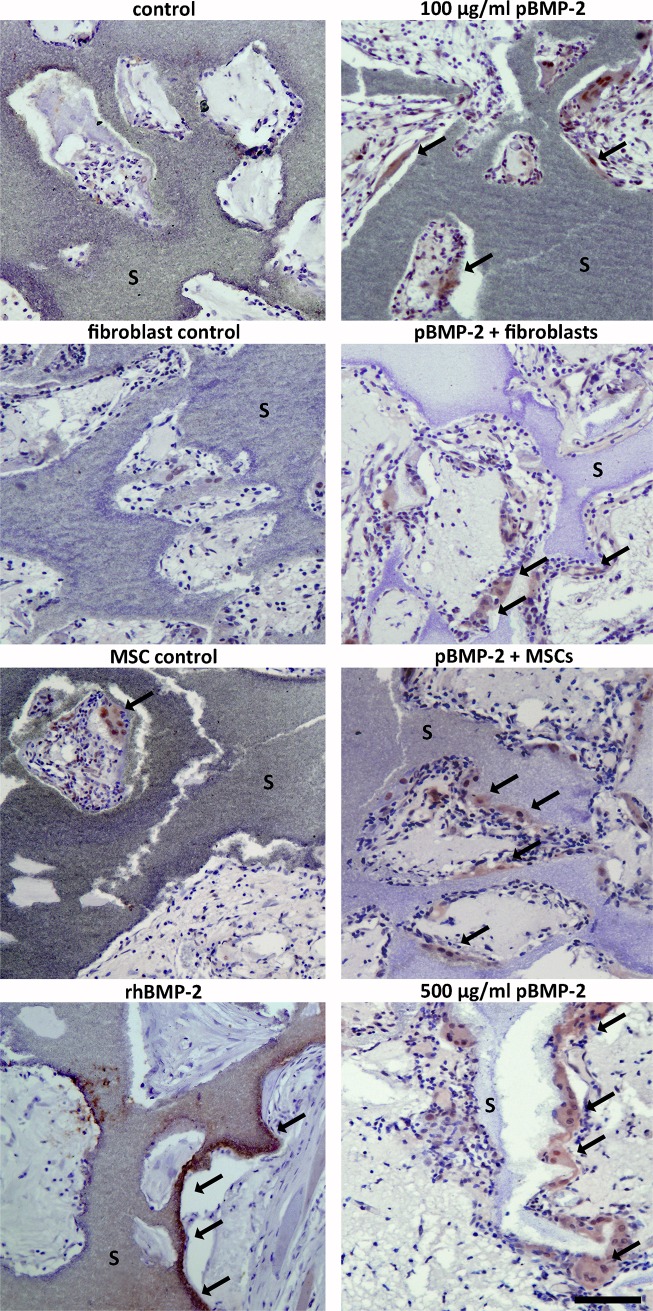
Identification of BMP-2 protein within the sample boundaries. A representative image of the BMP-2 immunohistochemistry is shown for each group. BMP-2 (brown staining, indicated with arrows) is present in all the groups containing pBMP-2, as well as the rhBMP-2 positive control group. The empty vector control groups showed BMP-2 presence sporadically, also when MSCs or fibroblasts were seeded. The BMP-2 antibody recognizes rat endogenous and transgene derived human BMP-2. s = scaffold. Scale bar = 100 μm for all panels.

As transgene expression was pDNA dependent but a plateau was not reached, a 5 fold higher dosage was included in the pBMP-2 expression study. Furthermore the i.m. location was selected because transgene was more frequently observed in that location. BMP-2 immunohistochemistry was performed to detect the presence of BMP-2 and localize the producing cells, both in and around the implants. The antibody used is human-specific but, as a result of highly conserved sequence, cross-reacts with rat BMP-2. BMP-2 was found in all groups and appeared to be mainly present in and directly around cells that align the BCP scaffold material (Fig 4). The expression of BMP-2 varied among the individual groups. BMP-2 presence was most obvious in the groups seeded with MSCs and 100 μg/ml pBMP-2 and in unseeded samples containing 500 μg/ml pBMP-2. The groups seeded with fibroblasts (100 μg/ml pBMP-2) and unseeded samples with 100 μg/ml pBMP-2 resulted in frequent BMP-2 positive cells. The positive control with rhBMP-2 showed positively stained osteoblasts bordering the newly formed bone. The negative control groups, both with and without seeded cells, only rarely showed BMP-2 positive cells.

**Fig 5:**
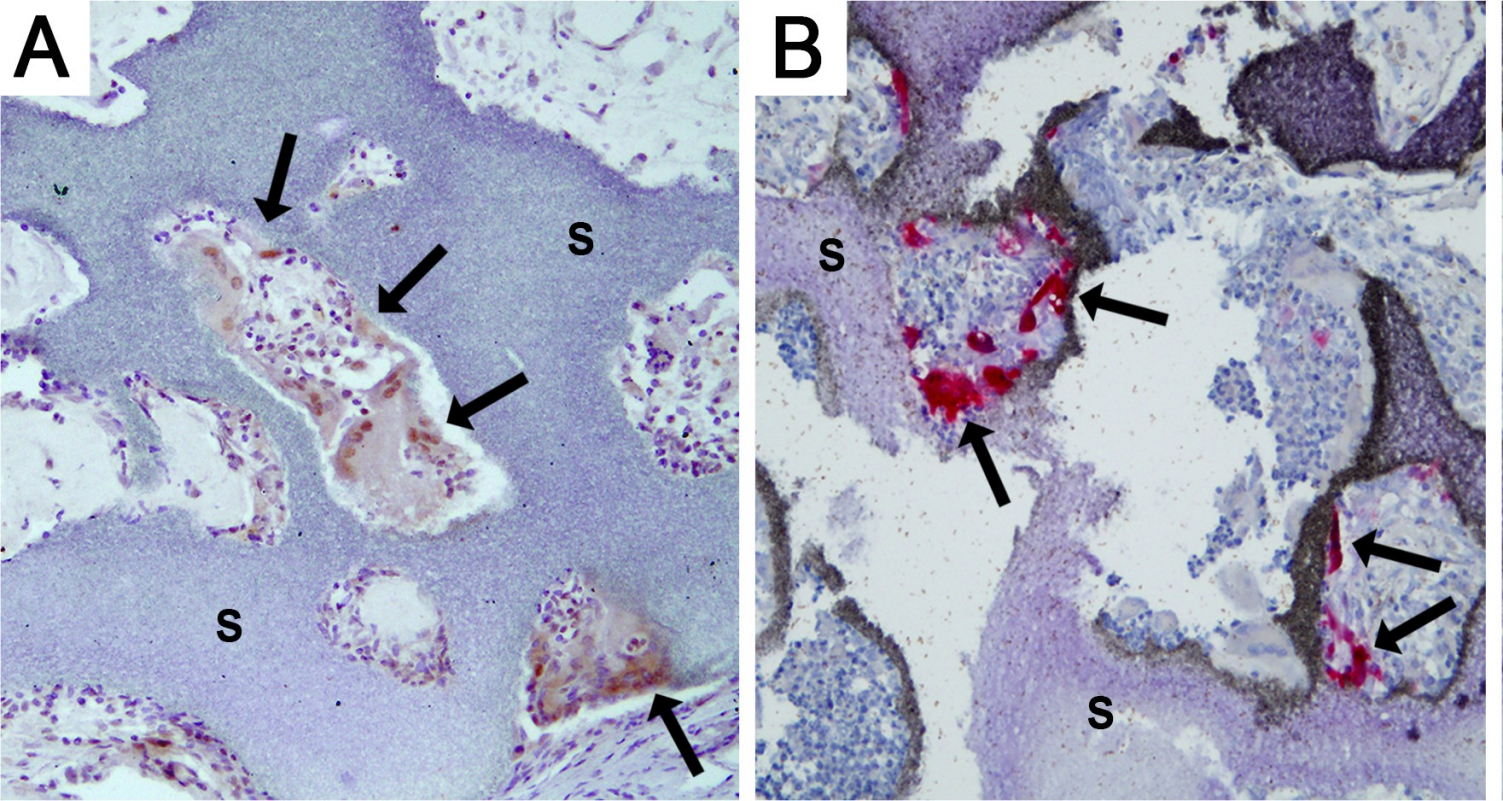
Characterization of the cells expressing BMP-2. BMP-2 immunohistochemistry (A) and TRAP staining for cells of the macrophage lineage (B) were performed on sections of the same group (pBMP-2 + MSCs). Cells aligning the scaffold, indicated with the arrows, are stained BMP-2 positive (brown in A) and TRAP positive (red in B). s = scaffold. Scale bar = 200 μm.

The MMA sections showed no bone formation in any of the groups with pBMP-2, whereas in seven out of eight rhBMP-2 containing constructs, bone was present. As we found plasmid-derived BMP-2 expression to be associated with TRAP-positive macrophophages in goat studies [33], we performed TRAP stainings on the rat intramuscular constructs. The positively stained multinucleated giant cells (MGC) were visible in regions directly contacting the scaffolds (Fig 5A), coinciding with the localization of BMP-2 positive cells (Fig 5B).

## Discussion

In current studies, the potentials and limitations of the alginate-based gene delivery method were investigated. The boundary conditions for efficiency of transgene expression using pGFP and pBMP-2 were determined *in vitro*. Then *in vivo* performance of cell-free and cell containing constructs elucidated that cell-free constructs loaded with pDNA in an alginate matrix can locally transfect the host cells. Furthermore, co-seeding of MSCs with this technique resulted in enhanced BMP-2 presence in the tissue. Despite evidence that local BMP-2 production was increased in the conditions with pBMP-2, this did not lead to ectopic bone formation. Detectible ectopic bone formation only occurred with the use of rhBMP-2.

To ensure adequate gene transfer and BMP-2 production in the *in vivo* bone-induction experiment, *in vitro* BMP-2 release was determined. Although fibroblasts and MSCs both produced BMP-2 after transfection, the average BMP-2 secretion was higher in MSCs. This could partly be explained by an autocrine feedback loop in MSCs, in which BMP-2 induces differentiation and endogenous BMP-2 production, as transfection efficiencies of MSCs and fibroblasts were similar (data not shown) [36]. Alternatively, the production capacity per cell type might differ.

The tracer gene GFP was used to assess transgene expression and to establish optimal plasmid DNA concentration *in vivo*. This marker is widely known for its sensitivity and great signal to noise ratio in tissue samples by immunohistochemistry. Using pGFP, the transfection of host cells was demonstrated after two weeks. Furthermore, the gene tracing experiment was set up to determine optimal plasmid DNA dosage. As a plateau effectivity of pDNA dosage could not be reached in the cell free groups and because high plasmid concentrations are not associated with severe side effects [34], we included one group with an even higher dose of pBMP-2 (500 μg/ml) in the bone-induction experiment.

To gain insight in the role of BMP-producing cells, MSCs and fibroblasts as producer cells were compared in plasmid-based gene delivery constructs. We hypothesized that fibroblasts could be suitable target cells for gene therapy, thereby functioning as a temporary BMP-2 delivery system. This role of fibroblasts stands in contrast to the role of MSCs, which function both as BMP-2 producer and subsequently become bone-forming cells. Although we found differential expression of BMP-2 in several groups, a clear correlation with bone formation was absent. The most obvious reason: the BMP production levels as a result of the *in vivo* transfection may have been insufficient. We showed that 10^6^ cells can in vitro produce up to 1 nanogram of BMP-2 after 7 days (Fig 1D). Unfortunately these results can not be directly translated to the *in vivo* situation, as local circumstances differ greatly. With the GFP tracer study we showed that adequate transfection takes place, but the precise amount of transgenic protein produced i*n vivo* can not be determined. GFP appears clearly in the immunohistochemistry due to the enhancement steps in the protocol, leading to an estimation of the number of cells transfected, not of the protein levels. Even if *in vivo* BMP production per cell is comparable to the *in vitro* production, it remains debatable whether it will be enough to result in bone formation in clinical relevant bone defects. As a comparison, viral transfection strategies have shown to reach up to 200 nanograms BMP per day per 10^6^ cells, resulting in spinal fusions in rats [37, 38].

To optimize host cell transfection, the timing of pDNA release together with the presence of host cells is essential [39]. From the present study it is unknown whether the release of plasmid DNA did or did not match with the presence and/or invasion of target cells. The first preclinical experiments with pDNA that applied a single extremely high dosage only resulted in a minimal increase of gene expression [34, 40]. Interestingly, repeated injections, ensuring prolonged presence of plasmid DNA, did show a strong positive effect on bone formation [41, 42]. Since we observed that pDNA is readily released from alginate *in vitro* and then becomes subject to washout, we suspect that a limited cellular invasion into the constructs was the cause of low BMP-2 expression and absence of bone formation in this group.

Despite the fact that the BMP-2 levels appeared too low to induce bone formation, our results indicate that cell seeding is not obligatory for transgene expression. Most of the BMP-2 expression was associated with multinucleated giant cells, even in the conditions with seeded target cells. Active degradation of the alginate/pDNA complex by the observed MGCs might have diminished the transfection and subsequent BMP-2 production of other resident cells (Fig 5), thus affecting the sustained pDNA release profile as observed *in vitro* (Fig 1B). We and others have shown that the application of genetically engineered MSCs could in some cases lead to long-term engraftment or result in temporary local BMP production, as the majority of the cells disappear from the implant area [25, 26]. Furthermore, it has been shown that bone formation as a result of viral BMP gene therapy seems to be independent of cell type [38]. These observations underline that the amount of BMP-2 production, together with its timing, are more essential to the process of bone formation than the presence of seeded bone-forming cells during gene delivery.

The exact role of alginate in the method of transfection has been extensively debated. The mechanism behind this transfection has not yet been elucidated, but all data obtained so far lead to the following theory. The alginate forms condensed complexes with pDNA and thus protects the DNA-structures from nucleases. Then, the alginate participates actively in pDNA delivery to the cytosol of cells. High extracellular calcium levels may stabilize the complexes which has shown to result in adequate gene delivery. Thirdly, pDNA release by alginate hydrogel into the surrounding tissue occurs in a timely fashion which results in a continuous controlled presence [43, 44].

In conclusion, the conditions for optimal transgene expression using the alginate gene activated matrix in rats were determined and secondly cell seeding is dispensable for non-viral BMP-2 gene expression *in vivo*. When investigating the role of BMP-producing cells, we found that bone progenitor cells outperformed non-osteogenic producer cells (i.e. fibroblasts) in plasmid-based gene delivery constructs. Despite optimization of current strategy, no bone was formed. Therefore we doubt whether this technique is robust enough to ensure adequate BMP production in clinical relevant models. Other strategies, including more efficient *ex-vivo* transfection methods and/or the use of hydrogel systems that allow cellular infiltration and show sustained release kinetics of plasmid DNA, might be interesting options for increasing efficacy in bone regeneration strategies.

## Supporting information

S1 Data Original data of graph depicted in Fig 1B (file Fig 1B DATA). (XLSX)

S2 Data Original data of graph depicted in Fig 1D (file Fig 1D DATA). (XLSX)

## References

[1] Friedenstein AJ, Piatetzky S II, Petrakova KV Osteogenesis in transplants of bone marrow cells. Journal of embryology and experimental morphology 1966; 16: 381–390 5336210

[2] Bruder SP, Fink DJ, Caplan AI Mesenchymal stem cells in bone development, bone repair, and skeletal regeneration therapy. J Cell Biochem 1994; 56: 283–294. 10.1002/jcb.240560303 7876320 PMC7166813

[3] Asatrian G, Pham D, Hardy WR, et al Stem cell technology for bone regeneration: current status and potential applications. Stem cells and cloning: advances and applications 2015; 8: 39–48. 10.2147/SCCAA.S48423 25709479 PMC4334288

[4] Becquart P, Cambon-Binder A, Monfoulet LE, et al Ischemia is the prime but not the only cause of human multipotent stromal cell death in tissue-engineered constructs in vivo. Tissue Eng Part A 2012; 18: 2084–2094. 10.1089/ten.TEA.2011.0690 22578283

[5] Dupont KM, Sharma K, Stevens HY, et al Human stem cell delivery for treatment of large segmental bone defects. Proc Natl Acad Sci U S A 2010; 107: 3305–3310. 10.1073/pnas.0905444107 20133731 PMC2840521

[6] Muschler GF, Nakamoto C, Griffith LG Engineering principles of clinical cell-based tissue engineering. J Bone Joint Surg Am 2004; 86-A: 1541–1558 10.2106/00004623-200407000-00029 15252108

[7] Dawson JI, Kanczler J, Tare R, et al Concise review: bridging the gap: bone regeneration using skeletal stem cell-based strategies—where are we now? Stem cells (Dayton, Ohio) 2014; 32: 35–44. 10.1002/stem.1559 24115290

[8] Grayson WL, Bunnell BA, Martin E, et al Stromal cells and stem cells in clinical bone regeneration. Nature reviews Endocrinology 2015; 11: 140–150. 10.1038/nrendo.2014.234 25560703 PMC4338988

[9] Glass GE, Jain A Cochrane corner: bone morphogenetic protein (BMP) for fracture healing in adults. The Journal of hand surgery, European volume 2013; 38: 447–449. 10.1177/1753193412474593 23612734

[10] McKay WF, Peckham SM, Badura JM A comprehensive clinical review of recombinant human bone morphogenetic protein-2 (INFUSE Bone Graft). International orthopaedics 2007; 31: 729–734. 10.1007/s00264-007-0418-6 17639384 PMC2266665

[11] Fu R, Selph S, McDonagh M, et al Effectiveness and harms of recombinant human bone morphogenetic protein-2 in spine fusion: a systematic review and meta-analysis. Ann Intern Med 2013; 158: 890–902. 10.7326/0003-4819-158-12-201306180-00006 23778906

[12] Poynton AR, Lane JM Safety profile for the clinical use of bone morphogenetic proteins in the spine. Spine 2002; 27: S40–S48. 10.1097/01.Brs.0000020734.34957.6a 12205419

[13] Geiger M, Li RH, Friess W Collagen sponges for bone regeneration with rhBMP-2. Adv Drug Deliv Rev 2003; 55: 1613–1629 14623404 10.1016/j.addr.2003.08.010

[14] Benglis D, Wang MY, Levi AD A comprehensive review of the safety profile of bone morphogenetic protein in spine surgery. Neurosurgery 2008; 62: ONS423–431; discussion ONS431. 10.1227/01.neu.0000326030.24220.d8 18596525

[15] Cho TJ, Gerstenfeld LC, Einhorn TA Differential temporal expression of members of the transforming growth factor beta superfamily during murine fracture healing. Journal of bone and mineral research: the official journal of the American Society for Bone and Mineral Research 2002; 17: 513–520. 10.1359/jbmr.2002.17.3.513 11874242

[16] Lissenberg-Thunnissen SN, de Gorter DJ, Sier CF, et al Use and efficacy of bone morphogenetic proteins in fracture healing. International orthopaedics 2011; 35: 1271–1280. 10.1007/s00264-011-1301-z 21698428 PMC3167450

[17] Dickerman RD, Reynolds AS, Morgan BC, et al rh-BMP-2 can be used safely in the cervical spine: dose and containment are the keys! Spine J 2007; 7: 508–509. 10.1016/j.spinee.2007.03.003 17521966

[18] Poldervaart MT, Wang H, van der Stok J, et al Sustained release of BMP-2 in bioprinted alginate for osteogenicity in mice and rats. PLoS One 2013; 8: e72610 10.1371/journal.pone.0072610 23977328 PMC3747086

[19] Evans CH, Huard J Gene therapy approaches to regenerating the musculoskeletal system. Nature reviews Rheumatology 2015; 11: 234–242. 10.1038/nrrheum.2015.28 25776949 PMC4510987

[20] Giacca M, Zacchigna S VEGF gene therapy: therapeutic angiogenesis in the clinic and beyond. Gene Ther 2012; 19: 622–629. 10.1038/gt.2012.17 22378343

[21] Wegman F, Bijenhof A, Schuijff L, et al Osteogenic differentiation as a result of BMP-2 plasmid DNA based gene therapy in vitro and in vivo. Eur Cell Mater 2011; 21: 230–242; discussion 242 21409753 10.22203/ecm.v021a18

[22] Virk MS, Sugiyama O, Park SH, et al "Same day" ex-vivo regional gene therapy: a novel strategy to enhance bone repair. Molecular therapy: the journal of the American Society of Gene Therapy 2011; 19: 960–968. 10.1038/mt.2011.2 21343916 PMC3098640

[23] Sheyn D, Pelled G, Zilberman Y, et al Nonvirally engineered porcine adipose tissue-derived stem cells: use in posterior spinal fusion. Stem cells (Dayton, Ohio) 2008; 26: 1056–1064. 10.1634/stemcells.2007-0858 18218819

[24] Geuze RE, Prins HJ, Oner FC, et al Luciferase labeling for multipotent stromal cell tracking in spinal fusion versus ectopic bone tissue engineering in mice and rats. Tissue Eng Part A 2010; 16: 3343–3351. 10.1089/ten.TEA.2009.0774 20575656

[25] Kruyt MC, Stijns MM, Fedorovich NE, et al Genetic marking with the DeltaLNGFR-gene for tracing goat cells in bone tissue engineering. J Orthop Res 2004; 22: 697–702. 10.1016/j.orthres.2003.10.021 S0736026603002729 [pii] 15183423

[26] Pensak M, Hong S, Dukas A, et al The role of transduced bone marrow cells overexpressing BMP-2 in healing critical-sized defects in a mouse femur. Gene Ther 2015 10.1038/gt.2015.14 25809463

[27] Evans CH Gene delivery to bone. Adv Drug Deliv Rev 2012; 64: 1331–1340. 10.1016/j.addr.2012.03.013 S0169-409X(12)00109-3 [pii] 22480730 PMC3392363

[28] Krebs MD, Salter E, Chen E, et al Calcium alginate phosphate-DNA nanoparticle gene delivery from hydrogels induces in vivo osteogenesis. Journal of Biomedical Materials Research Part A 2010; 92A: 1131–1138. 10.1002/Jbm.A.32441 19322877

[29] Kong HJ, Kim ES, Huang YC, et al Design of biodegradable hydrogel for the local and sustained delivery of angiogenic plasmid DNA. Pharm Res 2008; 25: 1230–1238. 10.1007/s11095-007-9526-7 18183476

[30] Park DJ, Choi JH, Leong KW, et al Tissue-engineered bone formation with gene transfer and mesenchymal stem cells in a minimally invasive technique. Laryngoscope 2007; 117: 1267–1271. 10.1097/MLG.0b013e31805f680e 17507830

[31] Hunt NC, Grover LM Cell encapsulation using biopolymer gels for regenerative medicine. Biotechnol Lett 2010; 32: 733–742. 10.1007/s10529-010-0221-0 20155383

[32] Loozen LD, Wegman F, Oner FC, et al Porous bioprinted constructs in BMP-2 non-viral gene therapy for bone tissue engineering. J Mater Chem B 2013; 1: 6619–6626. 10.1039/C3tb21093f 32261270

[33] Loozen L, Helm van der YJ, Oner FC, et al Bone morphogenetic protein-2 non-viral gene therapy in a goat iliac crest model for bone formation. Tissue Eng Part A 2015; 21: 1672–1679. 10.1089/ten.TEA.2014.0593 25719212

[34] Bonadio J, Smiley E, Patil P, et al Localized, direct plasmid gene delivery in vivo: prolonged therapy results in reproducible tissue regeneration. Nat Med 1999; 5: 753–759. 10.1038/10473 10395319

[35] Oddie GW, Schenk G, Angel NZ, et al Structure, function, and regulation of tartrate-resistant acid phosphatase. Bone 2000; 27: 575–584 11062342 10.1016/s8756-3282(00)00368-9

[36] Hsu WK, Wang JC, Liu NQ, et al Stem cells from human fat as cellular delivery vehicles in an athymic rat posterolateral spine fusion model. J Bone Joint Surg Am 2008; 90: 1043–1052. 10.2106/JBJS.G.00292 18451397

[37] Gugala Z, Davis AR, Fouletier-Dilling CM, et al Adenovirus BMP2-induced osteogenesis in combination with collagen carriers. Biomaterials 2007; 28: 4469–4479. 10.1016/j.biomaterials.2007.07.007 17645942

[38] Gugala Z, Olmsted-Davis EA, Gannon FH, et al Osteoinduction by ex vivo adenovirus-mediated BMP2 delivery is independent of cell type. Gene Ther 2003; 10: 1289–1296. 10.1038/sj.gt.3302006 12883525

[39] Yasuda K, Richez C, Uccellini MB, et al Requirement for DNA CpG content in TLR9-dependent dendritic cell activation induced by DNA-containing immune complexes. J Immunol 2009; 183: 3109–3117. 10.4049/jimmunol.0900399 19648272 PMC2860771

[40] Fang J, Zhu YY, Smiley E, et al Stimulation of new bone formation by direct transfer of osteogenic plasmid genes. Proc Natl Acad Sci U S A 1996; 93: 5753–5758 10.1073/pnas.93.12.5753 8650165 PMC39133

[41] Osawa K, Okubo Y, Nakao K, et al Osteoinduction by repeat plasmid injection of human bone morphogenetic protein-2. J Gene Med 2010; 12: 937–944. 10.1002/jgm.1515 21069645

[42] Feichtinger GA, Hofmann AT, Wassermann K, et al Constitutive and inducible co-expression systems for non-viral osteoinductive gene therapy. Eur Cell Mater 2014; 27: 166–184; discussion 184 24554273 10.22203/ecm.v027a13

[43] Lee KY, Mooney DJ Alginate: properties and biomedical applications. Progress in polymer science 2012; 37: 106–126. 10.1016/j.progpolymsci.2011.06.003 22125349 PMC3223967

[44] Jo J, Tabata Y. How controlled release technology can aid gene delivery. Expert Opin Drug Deliv. 2015;12(10):1689–701. 10.1517/17425247.2015.1048221 26119697

